# High Expression Levels of *ACTN1* and *ACTN3* Indicate Unfavorable Prognosis in Acute Myeloid Leukemia

**DOI:** 10.7150/jca.31766

**Published:** 2019-07-10

**Authors:** Xinrui Yang, Yifan Pang, Jilei Zhang, Jinlong Shi, Xinpei Zhang, Gaoqi Zhang, Siyuan Yang, Jing Wang, Kai Hu, Jijun Wang, Hongmei Jing, Xiaoyan Ke, Lin Fu

**Affiliations:** 1 Department of Hematology and Lymphoma Research Center, Peking University, Third Hospital, Beijing, 100191, China.; 2 Department of Medicine, William Beaumont Hospital, Royal Oak, MI 48073, USA.; 3 Department of Biomedical Engineering, Chinese PLA General Hospital, Beijing, 100853, China.

**Keywords:** acute myeloid leukemia, *ACTN1*, * ACTN3*, prognosis

## Abstract

**Background**: Actinins are major cytoskeletal proteins that mediate sarcomere function, and they also have important non-muscle functions such as regulating cytokinesis, cell adhesion and migration. There are four isoforms of actinins in mammals (ACTN1-4). Recently, the relationship between actinins and cancer has been discovered in many types of malignancy, yet their prognostic significance in acute myeloid leukemia (AML) remains unclear.

**Methods**: We collected data of 155 de novo AML patients from The Cancer Genome Atlas (TCGA) database; 85 patients received chemotherapy only and 70 patients underwent allogeneic hematopoietic stem cell transplantation (allo-HSCT). We divided each treatment groups into sub-groups based on the median expression levels of *ACTN1-4*.

**Results**: Survival analysis showed that in the chemotherapy-only group, high *ACTN1* and *ACTN3* expression were associated with shorter event-free survival (EFS) and overall survival (OS) (p<0.01). Multivariate analysis suggested that high expression of *ACTN1* and *ACTN3* (p<0.05) were independent poor prognostic factors. In the allo-HSCT group, *ACTN1-4* expression had no impact on survival.

**Conclusions**: Our study suggested that high expression levels of *ACTN1* and *ACTN3* adversely affected the survival of AML patients, but their harmful impact could be overcome by allo-HSCT.

## Background

Acute myeloid leukemia (AML) is a highly heterogeneous and aggressive malignancy of the undifferentiated or partially-differentiated bone marrow myeloid stem cells and progenitor cells^1^. In the past decade, next generation sequencing (NGS) technique has been widely used and became a powerful tool in AML research, greatly improving our understanding of the genetic basis of the disease. Many prognostic biomarkers were found with the help of NGS. For example, *NPM1* mutation and biallelic* CEBPA* mutations are favorable factors, associating with longer event-free survival (EFS) and overall survival (OS). On the other hand, the present of *FLT3-ITD* and *MLL-PTD,* mutations in *DNMT3A, RUNX1, TET2,* and *KRAS* are predictors for poor outcomes in AML patients^2^-^4^. In addition, aberrant epigenetic modification, i.e., dysregulated expression levels of certain genes, may also influence the prognosis.

Actinins are a group of cytoskeletal molecules that belongs to the actin filament cross-linking proteins^5^. There are four actinin isoforms in mammals, namely ACTN1-4. ACTN1 and ACTN4 are universally expressed in most tissues and cell types. ACTN2 are mainly expressed in the myocardium, skeletal muscle and brain. ACTN3 mostly appear in the skeletal muscle. In muscle cells, actinins link the adjacent sarcomeres together through thin filaments, to coordinate muscle contraction. In non-muscle cells, actinins also exhibit a myriad of functions^6^. Actinins participate in cytokinesis by balancing the contraction of myosin II, forming a contractile ring with the latter to eventually divide the mother cell in two. Actinins can format and disassemble cell-matrix adhesion through the activation of phosphoinositide 3- kinase (PI3K), or build different cell-cell adhesions by working with integrins and intercellular adhesion molecules (ICAMs). They are also indispensable for endocytosis and exocytosis, which are essential biological processes for neurons and synapses. ACTN4 has recently been found to be a transcriptional regulator.

Since ACTN4's prognostic value in breast cancer was established, many works have been done to investigate the role of actinins in cancer prognostication and tumorigenesis^7^. Increased expression of *ACTN4* has been associated with poor prognosis in ovarian cancer, colorectal cancer and acute lymphoblastic leukemia^8^-^10^. *ACTN1* is crucial for glioma cell motility, and it also plays an important role in lung adenocarcinoma^11^, ^12^. There are also studies pointing out the interactions between ACTN2 and ACTN3 with the parafibromin tumor suppressor protein, one of the proteins that is involved in the hypermethylation and suppression of many oncogenes^13^.

However, the impacts of ACTN1-4 on the clinical and biological features of AML, as well as their prognostic value, remain unclear. Herein, we analyzed the relationship between the expression levels of *ACTN1-4* and the outcomes of AML patients, hoping to guide future research in these areas.

## Methods

### Patients

We screened for de novo AML patients with *ACTN1-4* expression data at diagnosis from The Cancer Genome Atlas (TCGA) database. A total of 155 patients had available data for analysis. Among them, 85 patients received only chemotherapy (chemotherapy-only group) and 70 patients underwent allogeneic hematopoietic stem cell transplantation (allo-HSCT, allo-HSCT group). Baseline demographic, laboratory and genetic data were downloaded from the TCGA public website. Gene expression profiling was performed on the Affymetrix U133 plus 2 platform. The Clinical endpoints were event-free survival (EFS) and overall survival (OS). EFS is the time from diagnosis to removal from the study due to death, relapse, failure to achieve complete remission (CR), or censored at the last follow-up. OS is the time from diagnosis to death from any cause or censored at the last follow-up. Written informed consent was obtained from all patients, and the database was approved by the Human Research Ethics Committee of Washington University.

### Statistical analysis

The clinical and biological characteristics of patients were summarized by descriptive statistics. Numerical data was compared using the Mann-Whitney U test and categorical data was compared using the Chi-square test. Survival was depicted by the Kaplan-Meier method and compared by the log-rank test. Cox proportional hazard models were constructed for multivariate analysis in search of independent factors that influenced survival. Hazard ratios were presented with 95% confidence intervals. A two-sided P-value < 0.05 was considered statistically significant for all statistical analyses. All statistical analyses were performed by SPSS Version 20.0 software.

## Results

### Comparison of EFS and OS between different expression levels of *ACTN1-4*

**Figure 1:**
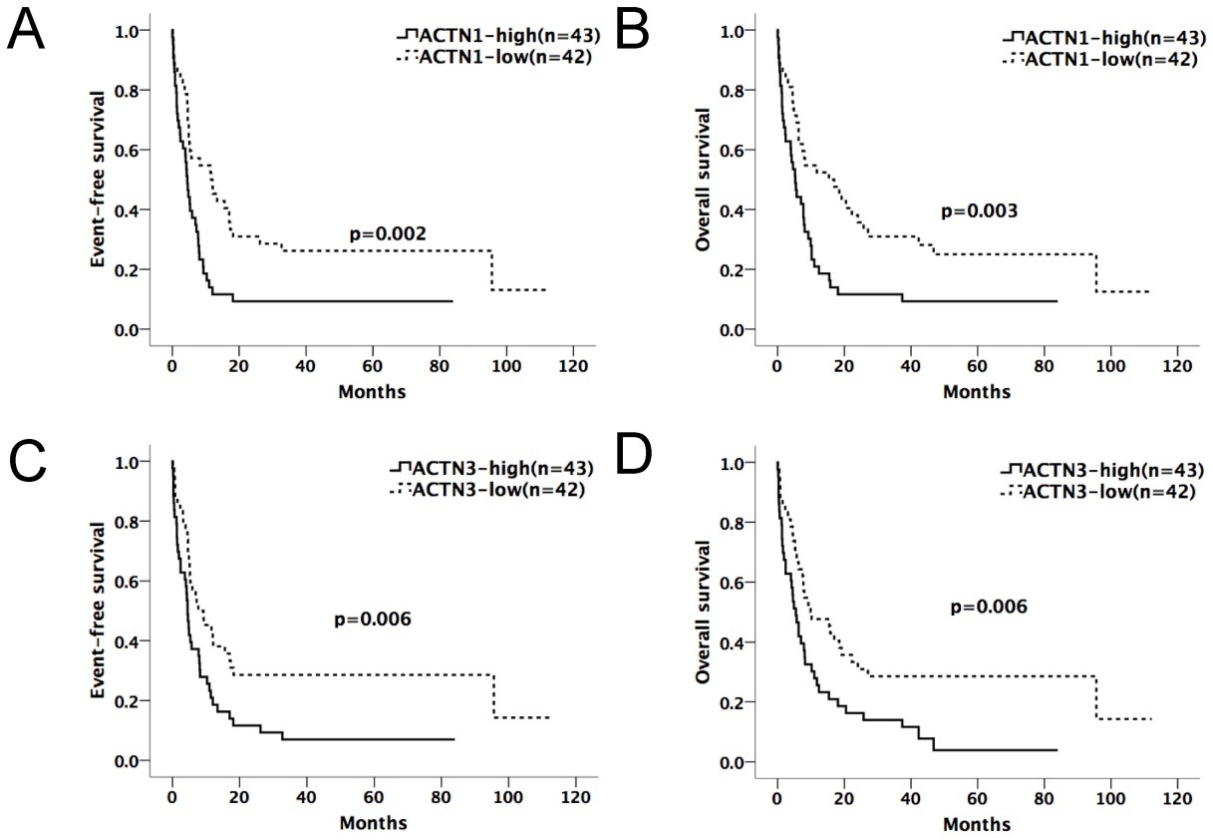
** Expression levels of *ACTN1*/*ACTN3* and patients' outcome in the chemotherapy-only group (n=85).** Patients with high expression levels of ACTN1 and ACTN3 had significantly shorter EFS (p=0.002, 0.006, respectively, Figures 1A and 1C) and OS (p=0.003, 0.006, respectively, Figures 1B and 1D).

We divided the chemotherapy-only group (n=85) and allo-HSCT group (n=70) patients into high and low-expression subgroups by their median *ACTN1-4* expression levels at diagnosis. *ACTN1-4* expression levels ≥ median was defined as high expression of the respective gene; others were defined as low expression. Kaplan-Meier method and the log-rank test were used for the analysis of EFS and OS between the high and low expression groups of each *ACTN* (Table 1A and 1B). In the chemotherapy-only group, patients with high expression levels of *ACTN1* and *ACTN3* had significantly shorter EFS (p=0.002, 0.006, respectively) and OS (p=0.003, 0.006, respectively), as shown in Figure 1A-D. *ACTN*2 was also influential (p=0.025 for EFS, p=0.039 for OS). However, *ACTN4* had no effect on EFS and OS. There was no statistical significance in survival between each *ACTN* high and low-expression subgroups in the allo-HSCT group.

### Association of clinical and biological characteristics with *ACTN1* or *ACTN3* expression levels

As demonstrated above, *ACTN1* and *ACTN3* had more significant impact on EFS and OS than *ACTN2* and *ACTN4*, hence we analyzed the association between the clinical, biological features and the expression levels of *ACTN1* (high vs. low) and *ACTN3* (high vs. low) in the entire cohort (n=155, Table 2). High *ACTN1* expression was associate with more patients over age 60 (p=0.037), lower WBC counts (p=0.010), fewer patients with FAB-M4 (p=0.032), more patients with complex karyotype (p=0.022) or poor-risk cytogenetics (p=0.001). Patient with higher *ACTN1* also had higher frequencies in *TP53* (p=0.005) and* WT1* (p=0.018). High *ACTN3* expression was associate with more patients > 60 years old, lower WBC counts, fewer patients with FAB-M4 subtype and more with FAB-M0 (p=0.002, p=0.002, p=0.018, and p=0.001, respectively). Similar to *ACTN1*, patients with high *ACTN3* expression also tended to have complex karyotype and poor-risk cytogenetics (both p=0.000). Fewer *FLT3* mutations (p=0.002), more frequent *IDH1/2* mutations (p=0.049) and *TP53* mutations (p=0.005) were found in high *ACTN3* expression patients.

### Prognostic impact of *ACTN1* and *ACTN3* expression in AML patients

**Figure 2:**
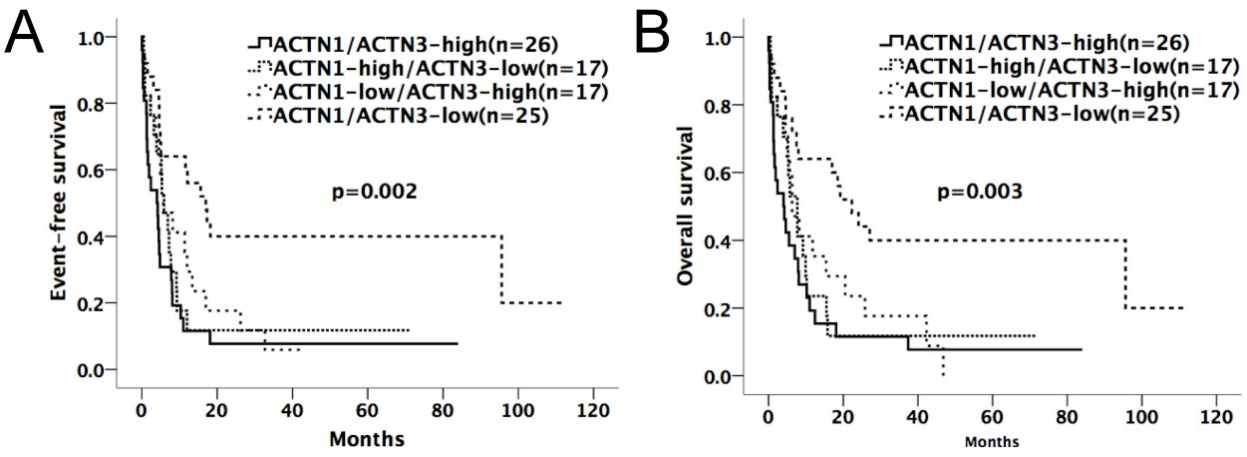
** Kaplan-Meier survival curves of different expression levels of *ACTN1/ACTN3* in the chemotherapy-only group.** Patients with high expressions of either *ACTN1* or *ACTN3* had shorter EFS and OS compared with those with low expressions of both (p=0.002 for EFS, p=0.003 for OS, Figure 2A and 2B).

**Figure 3:**
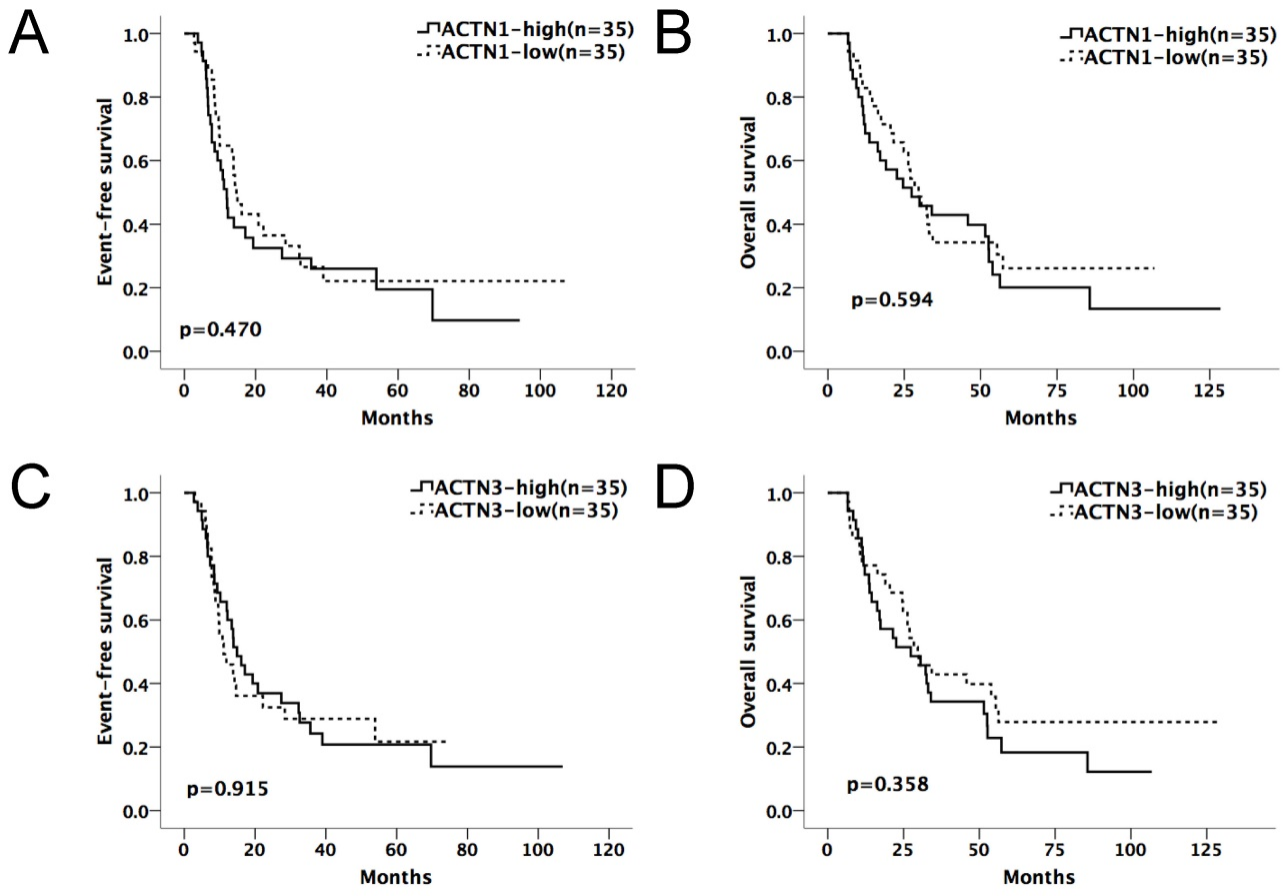
** Expression levels of *ACTN1*/*ACTN3* and patients' outcome in the allo-HSCT group.** No significant difference was found in EFS or OS comparing high and low expression groups of *ACTN1* and *ACTN3.* (p>0.05, Figure 3A and 3B).

Kaplan-Meier survival curves suggested that in the chemotherapy-only group, AML patients with high expressions of either *ACTN1* or *ACTN3* had shorter EFS and OS compared with patients with low expressions (p=0.002 for EFS, p=0.003 for OS, Figure 2A and 2B). However, no significant difference was found when doing similar comparison in the allo-HSCT group (p>0.05, Figure 3A and 3B).

Multivariate analysis was implemented to evaluate the prognostic value of clinical and biological variables in the chemotherapy-only group in order to avoid the influence of allo-HSCT. The expression levels of *ACTN1*,* ACTN2* and *ACTN3* (high vs. low), age (≥60 vs. <60 years), WBC count (≥15 vs. <15×109/L), *FLT3-ITD* (positive vs. negative) and other common AML mutations with relatively high frequency in this study (*NPM1*, *DNMT3A*, *IDH1/2*, *RUNX1*, *TET2* and *NRAS/KRAS*; mutated vs. wild type), were selected to construct the Cox regression model (Table 3). High expression levels of *ACTN1* (p=0.007 for EFS, p=0.021 for OS) and *ACTN3* (p=0.048 for EFS, p=0.018 for OS), older age (p=0.000 for EFS, p=0.002 for OS) independently contributed to poor prognosis.

## Discussion

Since Honda, et. al., 7 found that *ACTN4* was associated with poor prognosis in breast cancer patients, much laboratory and clinical work has been done to illustrate the relationship between actinins and solidary tumors, but little attention had been paid to that between actinins and hematological disorders. Our study was among the first to glimpse into the prognostic significance of *ACTN1-4* in AML patients. High expression levels of *ACTN1* and *ACTN3* acted as adverse factors for outcome in the chemotherapy-only group. However, in the allo-HSCT group, we did not observe any effect of *ACTN1* and *ACTN3* on the patients' survival.

*ACTN4* has been wildly studied for its function in tumors. Like *ACTN4*, *ACTN1* is expressed in most cell types. Given the many similarities in amino acid sequence and actin-binding properties, it would be possible for different ACTNs to have overlapping functions.^6^ ACTN1 participates in the assembly of F-actin at invadopodia, modulates cell adhesion through regulation of focal adhesion kinase-Src interaction^14^, ^15^. Increased level of ACTN1 in the cell promotes migration and loss of polarity by reorganizing the actin cytoskeleton and E-cadherin-based adhesions^16^. *ACTN3* is the “speed gene” that determines the performances of athletes, with 18% of the human population totally deficient in this gene^17^, but its functions in diseases have rarely been studied. The knocking-off of *ACTN3* in mouse muscle can increase the activity of aerobic metabolism and influence sarcomere composition in a dose-dependent fashion^18^. The oncogenic effect of *ACTN1* and the metabolic effect of *ACTN3* could help explain the findings in our study, though the clear pathophysiological mechanism requires careful laboratory experiments to further delineate.

As the role of actinins in cancer progression became clear in recent years, targeted treatment against actinins has been under development. Craig, et. al., used small interfering RNAs to reduce the expression of *ACTN1* in murine tumor cells. *ACTN1* silencing disrupted cancer cell adhesion to murine surgical wounds and thereby prolonged the tumor-free survival^19^. Previous studies also reported that transforming growth factor beta (TGF-β) could induce *ACTN1* mRNA expression, so targeting TGF-β would be an alternative way to suppress ACTN^20^, ^21^. Our study pointed out that *ACTN1* and *ACTN3* might also be potential targets in AML treatment.

High* ACTN1/3* expression was associated with some of the traditional AML risk factors, such as older age, complex karyotype, and poor-risk cytogenetics. Despite the associations, *ACTN1/3* expression independently contributed to the poor prognosis in AML patients receiving only chemotherapy. This highlights the fact that although AML prognostication is complex and many factors are intertwined, a single gene could still exert a strong impact.

Allo-HSCT is a powerful treatment of AML to and it can overcome the harmful effect of some high-risk molecular biomarkers^22^. In our study, the adverse effect of high expression levels of *ACTN1* and *ACTN3* was not observed in the allo-HSCT group, suggesting that allo-HSCT might surmount the adverse effect of *ACTN1* and *ACTN3* overexpression in AML patients.

## Conclusion

In conclusion, our study found that high expression levels of* ACTN1* and *ACTN3* at diagnosis indicated unfavorable outcome in AML patients. The pathophysiological mechanism behind this remained to be elucidated. In the future, *ACTN1* and* ACTN3* could be considered used as biomarkers and indicators for allo-HSCT in AML, if their significant prognostic value were to be confirmed in larger prospective cohorts.

## Figures and Tables

**Table 1A:** Comparison of EFS and OS between high and low expression levels of *ACTN1-4* (Chemotherapy-only, n=85)

Variables	EFS			OS	
	χ^2^	*P*-value		χ^2^	*P*-value
*ACTN1* (high vs. low)	9.331	0.002		8.756	0.003
*ACTN2* (high vs. low)	5.051	0.025		4.251	0.039
*ACTN3* (high vs. low)	7.542	0.006		7.571	0.006
*ACTN4* (high vs. low)	0.149	0.700		0.049	0.825

Abbreviations: EFS, event-free survival; OS, overall survival.

**Table 1B:** Comparison of EFS and OS between high and low expression levels of *ACTN1-4* (HSCT, n=70)

Variables	EFS			OS	
	χ^2^	*P*-value		χ^2^	*P*-value
*ACTN1* (high vs. low)	0.521	0.470		0.284	0.594
*ACTN2* (high vs. low)	0.042	0.838		0.164	0.685
*ACTN3* (high vs. low)	0.012	0.915		0.845	0.385
*ACTN4* (high vs. low)	0.106	0.744		0.096	0.757

Abbreviations: EFS, event-free survival; OS, overall survival.

**Table 2:** Clinical and molecular characteristics of patients with high or low *ACTN1* and *ACTN3* expression levels

Characteristics	*ACTN1*				*ACTN3*		
	High (n=78)	Low (n=77)	*P*		High (n=78)	Low (n=77)	*P*
Age/years, median (range)	61 (18-81)	57 (22-88)	0.126^*^		62.5 (21-88)	56 (18-82)	0.007^*^
Age group/n (%)			0.037^§^				0.002^§^
< 60 years	32 (41.0)	45 (58.4)			29 (37.2)	48 (62.3)	
≥ 60 years	46 (59.0)	32 (41.6)			49 (62.8)	29 (37.7)	
Gender/n (%)			0.335^§^				0.108^§^
Male	46 (59.0)	39 (50.6)			48 (61.5)	37 (48.1)	
Female	32 (41.0)	38 (49.4)			30 (38.5)	40 (51.9)	
Race/n (%)			0.588^§^				0.275^§^
Caucasian	59 (75.6)	55 (71.4)			54 (69.2)	60 (77.9)	
Others	19 (24.4)	22 (28.6)			24 (30.8)	17 (22.1)	
WBC/×10^9^/L, median (range)	12.7 (0.6-171.9)	33.2 (1.0-297.4)	0.010^*^		11.1 (0.6-297.4)	33.2 (1.2-223.8)	0.002^*^
BM blast/%, median (range)	70.0 (30-100)	73 (33-99)	0.427^*^		69 (30-99)	72 (32-100)	0.893^*^
PB blast/%, median (range)	32 (0-97)	48 (0-98)	0.076^*^		32 (0-98)	47 (0-97)	0.349^*^
FAB subtypes/n (%)							
M0	6 (7.7)	10 (13.0)	0.304^§^		14 (17.9)	2 (2.6)	0.001^§^
M1	24 (30.8)	19 (24.7)	0.473^§^		21 (26.9)	22 (28.6)	1.000^§^
M2	23 (29.5)	16 (20.8)	0.266^§^		18 (23.1)	21 (27.3)	0.711^§^
M4	11 (14.1)	22 (28.6)	0.032^§^		10 (12.8)	23 (29.9)	0.018^§^
M5	10 (12.8)	7 (9.1)	0.608^§^		9 (11.5)	8 (10.4)	0.803^§^
M6	1 (1.3)	1 (1.3)	1.000^§^		2 (2.6)	0 (0.0)	0.245^§^
M7	2 (2.6)	1 (1.3)	1.000^§^		2 (2.6)	1 (1.3)	0.620^§^
No date	1 (1.3)	1 (1.3)			2 (2.6)	0 (0.0)	
Karyotype/n (%)							
Normal	36 (46.2)	37 (48.1)	0.871^§^		31 (39.7)	42 (54.5)	0.108^§^
Complex	17 (21.8)	6 (7.8)	0.022^§^		20 (25.6)	3 (3.9)	0.000^§^
8 Trisomy	5 (6.4)	2 (2.6)	0.442^§^		6 (7.7)	1 (1.3)	0.062^§^
inv(16)/CBFβ-MYH11	1 (1.3)	10 (13.0)	0.004^§^		0 (0.0)	11 (14.3)	0.001^§^
11q23/MLL	5 (6.4)	1 (1.3)	0.210^§^		1 (1.3)	5 (6.5)	0.210^§^
-7/7q-	5 (6.4)	1 (1.3)	0.210^§^		6 (7.7)	0 (0.0)	0.013^§^
t(9;22)/BCR-ABL1	2 (2.6)	1 (1.3)	1.000^§^		2 (2.6)	1 (1.3)	0.618^§^
t(8;21)/RUNX1-RUNX1T1	1 (1.3)	6 (7.8)	0.062^§^		1 (1.3)	6 (7.8)	0.117^§^
Others	5 (6.4)	11 (14.3)	0.118^§^		8 (10.3)	8 (10.4)	1.000^§^
No date	1 (1.3)	2 (2.6)			3 (3.8)	0 (0.0)	
Risk/n (%)							
Good	2 (2.6)	16 (20.8)	0.000^§^		1 (1.3)	17 (22.1)	0.000^§^
Intermediate	45 (57.7)	48 (62.3)	0.509^§^		43 (55.1)	50 (64.9)	0.406^§^
Poor	30 (38.5)	11 (14.3)	0.001^§^		31 (39.7)	10 (13.0)	0.000^§^
No date	1 (1.3)	2 (2.6)			3 (3.8)	0 (0.0)	
*MLL-PTD*			0.167^§^				0.495^§^
Presence	7 (9.0)	2 (2.6)			6 (7.7)	3 (3.9)	
Absence	71 (91.0)	75 (97.4)			72 (92.3)	74 (96.1)	
*FLT3*/n (%)			0.474^§^				0.002^§^
*FLT3*-ITD	17 (21.8)	14 (18.2)			10 (12.8)	21 (27.3)	
*FLT3*-TKD	7 (9.0)	5 (6.5)			3 (3.8)	9 (11.7)	
Wild type	54 (69.2)	58 (75.3)			65 (83.3)	47 (61.0)	
*NPM1*/n (%)			0.599^§^				0.599^§^
Mutation	21 (26.9)	24 (31.2)			21 (26.9)	24 (31.2)	
Wild type	57 (73.1)	53 (68.8)			57 (73.1)	53 (68.8)	
*DNMT3A*/n (%)			1.000^§^				0.583^§^
Mutation	20 (25.6)	20 (26.0)			22 (28.2)	18 (23.4)	
Wild type	58 (74.4)	57 (74.0)			56 (71.8)	59 (76.6)	
*IDH1*/*IDH2*/n (%)			0.846^§^				0.049^§^
Mutation	16 (20.5)	17 (22.1)			22 (28.2)	11 (14.3)	
Wild type	62 (79.5)	60 (77.9)			56 (71.8)	66 (85.7)	
*RUNX1*/n (%)			0.121^§^				0.429^§^
Mutation	5 (6.4)	11 (14.3)			10 (12.8)	6 (7.8)	
Wild type	73 (93.6)	66 (85.7)			68 (87.2)	71 (92.2)	
*TET2*/n (%)			0.588^§^				0.588^§^
Mutation	9 (11.5)	6 (7.8)			9 (11.5)	6 (7.8)	
Wild type	69 (88.5)	71 (92.2)			69 (88.5)	71 (92.2)	
*TP53*/n (%)			0.005^§^				0.001^§^
Mutation	13 (16.7)	2 (2.6)			14 (17.9)	1 (1.3)	
Wild type	65 (83.3)	75 (97.4)			64 (82.1)	76 (98.7)	
*NRAS*/*KRAS*/n (%)			0.811^§^				1.000^§^
Mutation	9 (11.5)	10 (13.0)			10 (12.8)	9 (11.7)	
Wild type	69 (88.5)	67 (87.0)			68 (87.2)	68 (88.3)	
*CEBPA*/n (%)			0.765^§^				0.368^§^
Single Mutation	5 (6.4)	3 (3.9)			4 (5.1)	4 (5.2)	
Double Mutation	0 (0.0)	3 (3.9)			0 (0.0)	3 (3.9)	
Wild type	73 (93.6)	71 (92.2)			74 (94.9)	70 (90.9)	
*WT1*/n (%)			0.018^§^				0.534^§^
Mutation	9 (11.5)	1 (1.3)			4 (5.1)	6 (7.8)	
Wild type	69 (88.5)	76 (98.7)			74 (94.9)	71 (92.2)	
*PTPN11*/n (%)			0.719^§^				0.495^§^
Mutation	5 (6.4)	3 (3.9)			3 (3.8)	5 (6.5)	
Wild type	73 (93.6)	74 (96.1)			75 (96.2)	72 (93.5)	
Relapse/n (%)			0.265^§^				0.426^§^
Yes	36 (46.2)	42 (54.5)			37 (47.4)	41 (53.2)	
No	42 (53.8)	34 (44.2)			41 (52.6)	35 (45.5)	
No date	0 (0.0)	1 (1.3)			0 (0.0)	1 (1.3)	

Abbreviations: WBC: white blood cell; BM: bone marrow; PB: peripheral blood; FAB: French American British. '*' denotes Mann-Whitney *U* test; '§' denotes chi-square test.

**Table 3:** Multivariate analysis for EFS and OS in the chemotherapy-only group

Variables	EFS			OS	
	HR (95%CI)	*P*-value		HR (95%CI)	*P*-value
*ACTN1* (high vs. low)	2.310 (1.257-4.245)	0.007		2.020 (1.110-3.673)	0.021
*ACTN2* (high vs. low)	1.679 (0.976-2.890)	0.061		1.519 (0.887-2.601)	0.128
*ACTN3* (high vs. low)	1.800 (1.005-3.225)	0.048		2.067 (1.134-3.768)	0.018
Age (≥60 vs. <60 years)	3.329 (1.732-6.400)	0.000		2.819 (1.486-5.348)	0.002
WBC (≥15 vs. <15×10^9^/L)	1.701 (0.951-3.040)	0.073		1.590 (0.903-2.800)	0.108
*FLT3-ITD* (positive vs. negative)	1.016 (0.505-2.043)	0.964		1.000 (0.485-2.061)	1.000
*NPM1* (mutated vs. wild)	1.293 (0.630-2.651)	0.484		0.948 (0.470-1.912)	0.882
*DNMT3A* (mutated vs. wild)	1.507 (0.795-2.857)	0.209		1.786 (0.946-3.372)	0.073
*IDH1/2* (mutated vs. wild)	0.507 (0.244-1.051)	0.068		0.526 (0.257-1.076)	0.079
*RUNX1* (mutated vs. wild)	2.353 (0.903-6.134)	0.080		2.446 (0.942-6.347)	0.066
*TET2* (mutated vs. wild)	0.542 (0.240-1.225)	0.141		0.363 (0.155-0.846)	0.019
*NRAS/KRAS* (mutated vs. wild)	0.663 (0.301-1.461)	0.308		0.812 (0.373-1.768)	0.600

Abbreviations: EFS, Event-free survival; OS, Overall survival; WBC, white blood cell; HR, hazard ratio; CI, confidence interval.

## Abbreviations

ACTN: actinin
ALL: acute lymphoblastic leukemia
AML: acute myeloid leukemia
CR: complete remission
EFS: event-free survival
HSCT: hematopoietic stem cell transplantation
ICAMs: intercellular adhesion molecules
NGS: next generation sequencing
OS: overall survival
PI3K: phosphoinositide 3- kinase
